# Hepcidin Levels and Their Determinants in Different Types of Myelodysplastic Syndromes

**DOI:** 10.1371/journal.pone.0023109

**Published:** 2011-08-19

**Authors:** Valeria Santini, Domenico Girelli, Alessandro Sanna, Nicola Martinelli, Lorena Duca, Natascia Campostrini, Agostino Cortelezzi, Michela Corbella, Alberto Bosi, Gianluigi Reda, Oliviero Olivieri, Maria Domenica Cappellini

**Affiliations:** 1 Hematology Unit, AOU Careggi, University of Florence, Florence, Italy; 2 Department of Medicine, Section of Internal Medicine, University of Verona, Verona, Italy; 3 Department of Internal Medicine, “Cà Granda” Foundation IRCCS, University of Milan, Milan, Italy; 4 Hematology Unit 1, “Cà Granda” Foundation IRCCS, University of Milan, Milan, Italy; University of Minnesota, United States of America

## Abstract

Iron overload may represent an additional clinical problem in patients with Myelodysplastic Syndromes (MDS), with recent data suggesting prognostic implications. Beyond red blood cells transfusions, dysregulation of hepcidin, the key iron hormone, may play a role, but studies until now have been hampered by technical problems. Using a recently validated assay, we measured serum hepcidin in 113 patients with different MDS subtypes. Mean hepcidin levels were consistently heterogeneous across different MDS subtypes, with the lowest levels in refractory anemia with ringed sideroblasts (RARS, 1.43 nM) and the highest in refractory anemia with excess blasts (RAEB, 11.3 nM) or in chronic myelomonocytic leukemia (CMML, 10.04 nM) (*P* = 0.003 by ANOVA). MDS subtypes remained significant predictors of hepcidin in multivariate analyses adjusted for ferritin and transfusion history. Consistently with current knowledge on hepcidin action/regulation, RARS patients had the highest levels of toxic non-transferrin-bound-iron, while RAEB and CMML patients had substantial elevation of C-Reactive Protein as compared to other MDS subtypes, and showed lost of homeostatic regulation by iron. Growth differentiation factor 15 did not appear as a primary hepcidin regulator in this series. If confirmed, these results may help to calibrate future treatments with chelating agents and/or hepcidin modulators in MDS patients.

## Introduction

Myelodysplastic syndromes (MDS) are a heterogeneous group of clonal stem cell disorders characterized by dysplastic and ineffective hematopoiesis, peripheral cytopenias often including severe anemia, and a variable risk of progression to acute myleogenous leukemia (AML) [1].

Iron overload frequently occurs in MDS patients [2], with recent data suggesting an impact on both overall and leukemia-free survival [3]. Though prolonged red blood cells (RBC) transfusion therapy appears the main contributor, many patients appear to develop iron overload at an early stage of the disease, before the onset of transfusions [4]. It has been postulated that an altered production of hepcidin, the recently discovered key hormone regulating iron homeostasis [5], may play a role at this regard [6]. Hepcidin is a small peptide that acts by binding to ferroportin, its receptor highly expressed on the membrane of cells involved in iron handling like iron absorbing duodenal enterocytes and macrophages recycling senescent erythrocytes [7]. Ferroportin, the only known cellular iron exporter in vertebrates, is internalized and degraded after hepcidin binding [8], which results in blocking both dietary iron absorption and the release of iron from macrophages. The regulation of hepcidin is complex and mediated by different stimuli with opposing effects [5], [9]. Increased hepatic and plasma iron homeostatically induce hepcidin synthesis, as does inflammation, while erythropoietic activity suppresses the hormone production [9]. The latter is finalized to increase iron supply for erythropoiesis through enhanced iron absorption and release from macrophages. Such effect becomes particularly important in diseases with ineffective erythropoiesis, where erythrocyte precursors massively expand but undergo apoptosis rather than maturing. Growth differentiation factor 15 (GDF-15), a protein produced by erythroid precursors, has been proposed to be a major hepcidin suppressor in β-thalassemia [10], but data in other conditions with ineffective erythropoiesis are less conclusive [11].

Until recently, clinical studies on hepcidin in humans have been hampered by problems in the development of reliable assays [12], [13]. Regarding MDS, only scanty and conflicting data based on first generation semi-quantitative measurement of urinary hepcidin have been reported [14], [15]. We used a recently validated and improved Mass-Spectrometry based method to analyze serum hepcidin levels in MDS patients, also focusing in trying to elucidate its determinants.

## Methods

### Patients

Patients and controls were enrolled at Internal Medicine and Hematology Units in Verona (Azienda Integrata Ospedaliera-Universitaria), Florence (Ospedale Careggi) and Milan (Policlinico), all in Italy. One hundred and thirteen MDS patients (mean age 72.8±9.2 years; 68.1% males) were included. To be enrolled in this study, patients had to be previously untreated or treated only with transfusions. Patients treated at any time with iron chelating agents were excluded. After careful evaluation of transfusion history, patients were defined transfusion-dependent or transfusion-independent according to International Working Group (IWG) criteria [16]. Reliable data in this sense were available for 107/133 patients. MDS subtypes were classified according to World Health Organization (WHO) [17], and stratified for prognosis according to International Prognostic Scoring System (IPSS). To do a comparison with respect to serum hepcidin levels and the hepcidin/ferritin ratio (see below), a group of fifty-four healthy individuals (61.1% males) with rigorous definition of normal iron status as previously described in details [18], [19], were used as controls. The protocol of this observational study was approved by the Ethical Committee of the Azienda Integrata Ospedaliera Universitaria of Verona, and all subjects gave written informed consent.

### Biochemical Assays

Blood samples were obtained early in the morning after overnight fasting, immediately centrifuged, and serum was stored at −80°C in aliquots to avoid multiple freeze-thaw cycles. Serum iron, transferrin, ferritin, and C-Reactive Protein (CRP) were measured using routine standard laboratory assays.

Serum hepcidin was measured by Surface-Enhanced Laser Desorption/Ionization Time-Of-Flight Mass Spectrometry (SELDI-TOF MS), using a synthetic hepcidin analogue (Hepdicin-24, Peptides International, Louisville, KY) as an internal standard, as previously described [19], with recent technical advances [20]. The ratio between hepcidin and ferritin, which reflects the homeostatic ability of hepcidin to increase as a response to increased body iron was calculated as previously described [21].

Serum non-transferrin-bound iron (NTBI) was evaluated by chromatographic method as previously described [22]. Briefly, 450 µl of serum was added to 50 µl of nitrilotriacetic acid 800 mM (pH 7.0) and allowed to stand for 15 min. The solution was ultrafiltered using an Amicon Centricon 30 microconcentrator and the ultrafiltrate (20 µl) was injected directly into the high performance liquid chromatography (HPLC) system with a titanium pump module (Perkin Elmer S200, Boston, MA, USA). The HPLC column used for the determination of NTBI had the following characteristics: Nova-Pak C18, 4 µm, 3.9×150 mm, reversed-phase column produced by Waters (Wexford, Ireland). The chromatographic conditions were the following: Flow rate 1.5 ml/min; mobile phase isocratic containing 20% acetonitrile and 80% sodium phosphate buffer, 5 mM (pH 7.0) containing 3 mM CP22; visible detection, 450 nm. A standard curve was generated by injecting different concentrations of iron prepared in a 100-fold excess of NTA. The standards were routinely run at 0 to 10 mM, although absorbance was linear up to 40 mM. Under these conditions, the 0 mM standard corresponds to 80 mM of NTA. The addition of 80 mM of NTA to the serum of normal individuals always results in negative NTBI values. Normal individuals always have negative NTBI values because blank is formed by water and nitrilotriacetic acid; water *per se* contains small amounts of iron that is not bound by transferrin, whereas in samples, transferrin, which is not completely saturated, captures some iron from the ferritin-nitrilotriacetic acid complex [23].

Serum GDF-15 was determined using a commercially available Duo-Set enzyme-linked immunosorbent assay (ELISA) according to manufacturer's indications (R&D Systems, Abingdon, Oxfordshire, UK).

Serum erythropoietin (EPO) was determined using a commercially available radioimmunoassay (EPO-Trac ^125^I) kit according to manufacturer's indications (DiaSorin, Stillwater, Minnesota, USA).

### Statistical analyses

All calculations were performed using SPSS 17.0 software (SPSS Inc., Chicago, IL, USA). As many of the continuous variables of interest, including serum hepcidin, ferritin, GDF-15, and EPO, showed a non-Gaussian distribution, their values were log-transformed and expressed as geometric means with 95% confidence intervals (CIs). Quantitative data were analysed using the Student's t test or by analysis of variance (ANOVA) with polynomial contrast for linear trend, when appropriate. Qualitative data were analyzed with the χ^2^ test and with χ^2^ analysis for linear trend, when appropriate. Correlations between quantitative variables were assessed using Pearson's coefficient. Independent determinants of serum hepcidin levels were assessed at first in a linear regression model estimating β-coefficients including all the variables significantly correlated with hepcidin at univariate analysis (e.g. ferritin, CRP), as well as age, gender, and presence/absence of the diagnosis of MDS. Thereafter, aiming to evaluate the potential heterogeneity in iron/hepcidin homeostasis among the different types of MDS, the latter were codified as dummy variables in linear regression models, adjusted at first for sex, age, history of blood transfusion, and ferritin levels (model 1), then adding CRP levels (model 2). To evaluate the different degree of correlation between hepcidin and ferritin among the different types of MDS, data were analyzed in a general linear model by means of the F test for slopes. Two-sided p values<0.05 were considered statistically significant.

## Results

### MDS patients versus controls

Table 1 shows the main characteristics and iron biochemical parameters including serum hepcidin of the whole MDS population as compared with the control group. Controls were matched for gender (predominantly males), but were significantly younger than MDS patients. Recent data from our laboratory in a large population study have shown that serum hepcidin levels are consistently stable in males over a wide age range (18–90 years) [24]. Nevertheless, all multivariate analyses on serum hepcidin levels were adjusted for gender. As shown in Table 1, biochemical markers of iron overload (namely serum ferritin and transferrin saturation), as well as CRP levels, were significantly higher in MDS patients as compared to controls. In the whole MDS population serum hepcidin levels were slightly higher than in controls, but this difference did not reach the statistical significance. Nevertheless, the hepcidin/ferritin ratio was significantly lower in the whole MDS population as compared to controls. As regards to serum GDF-15, we could not directly measure this protein in controls, but levels in MDS patients (4,422 pg/ml, 95% CIs 3,591–5,445) were markedly higher than the manufacturer's reference range (641 pg/ml, 95% CIs 401–881). The same was true also for serum NTBI and EPO levels (showed in Table S1).

**Table 1:** General characteristics of the whole MDS population as compared to a reference group with normal serum iron indices and hepcidin.

	Controls (n = 54)	MDS (n = 113)	*P*
**Age (years)**	34.8±15.8	72.8±9.2	<0.001
**Male sex (%)**	61.1	68.1	0.370
**CRP (mg/l)**	1.07 (0.93–1.23)	3.81(2.71–5.36)	<0.001
**Ferritin** * **(µg/l)**	79 (64–97)	515 (407–652)	<0.001
**Hepcidin** * **(nM/l)**	4.20 (3.53–5.00)	5.31 (3.98–7.08)	0.288
**Hepcidin/Ferritin Ratio** *	52.94 (43.57–64.33)	10.10 (7.53–13.53)	<0.001
**s-Iron (µg/dl)**	100±28	127±59	<0.001
**s-Transferrin (g/l)**	2.51±0.37	2.01±0.40	<0.001
**Transferrin Saturation (%)**	28.9±9.1	49.8±27.4	<0.001

*: geometric means with 95% Confidence Intervals between bracke.

### Transfusion dependent versus transfusion independent MDS patients

Biochemical parameters of MDS patients stratified according to the presence or absence of transfusion dependence are shown in Table S1. As expected, transfusion dependent (TD) MDS patients had significantly higher levels of serum ferritin and transferrin saturation as compared to transfusion independent patients. Notably, serum hepcidin levels were significantly higher in TD MDS patients as compared to either controls or non-TD patients, but the hepcidin/ferritin ratio was similar in TD and non-TD MDS patients. TD MDS patients had also significantly higher levels of serum NTBI, GDF-15, and EPO as compared to non-TD patients.

### MDS patients stratified according to different WHO subtypes

Table 2 shows the clinical and biochemical characteristics of the MDS patients stratified according to the WHO classification. Serum hepcidin levels showed a significant variability across the different MDS subtypes (P = 0.003 by ANOVA), with the lowest values in patients with refractory anemia with ringed sideroblasts (RARS) and the highest values in subjects with refractory anemia with excess blasts (RAEB) and in patients with chronic myelomonocytic leukemia (CMML). The hepcidin/ferritin ratio (also showed in Figure S1) was also markedly heterogeneous across the different MDS subtypes. It was remarkably lower not only in RARS but also in patients with the 5q- syndrome, while CMML patients showed the highest values (P = 0.003 by ANOVA). Of note, patients with RARS and with the 5q- syndrome also appeared as the most iron overloaded, as suggested by the trend toward higher levels not only of serum ferritin, but also of serum transferrin saturation (P = 0.048 by ANOVA) and serum NTBI. As regards to serum GDF-15 levels, they were consistently homogeneous across the different MDS subtypes (P = 0.976 by ANOVA). GDF-15 did not correlate at all with hepcidin levels (r = −0.07; P = 0.48). On the contrary, CRP levels were significantly heterogeneous in different MDS subtypes (P = 0.008 by ANOVA), with the highest values in patients with RAEB, CMML and in those unclassified. Table S2 shows the biochemical parameters in MDS patients stratified according to the IPSS.

**Table 2:** Clinical and biochemical characteristics of MDS patients stratified according to WHO classification system.

	RA (n = 31)	RARS (n = 9)	RCMD (n = 19)	RAEB (n = 32)	5q- syndrome (n = 7)	CMML (n = 7)	Unclass (n = 8)	*P* °
**Age (years)**	75.8±10.3	73.4±7.7	73.7±7.1	70.2±7.6	71.4±12.0	73.0±8.2	67.1±13.0	0.163
**Male sex (%)**	61.3	44.4	73.7	78.1	57.1	85.7	62.5	0.397
**CRP** * **(mg/l)**	2.09 (1.07–4.10)	1.46 (0.36–5.56)	3.30 (1.37–7.97)	9.13 (5.41–15.39)	2.21 (0.53–9.13)	5.03 (0.19–129.90)	10.77 (5.16–22.48)	0.008
**Ferritin** * **(µg/l)**	368 (231–586)	725 (403–1305)	420 (230–768)	661 (461–947)	1364 (233–8001)	289 (130–646)	580 (135–2493)	0.104
**Hepcidin** * **(nmol/l)**	3.46 (2.06–5.81)	1.43 (0.51–4.03)	3.83 (1.85–7.96)	11.31 (7.38–17.32)	6.62 (1.26–34.84)	10.04 (2.10–48.00)	6.06 (1.18–31.27)	0.003
**Hepcidin/Ferritin** * **Ratio (nmol/µg×1000)**	9.39 (5.72–15.41)	1.97 (0.86–4.52)	9.13 (3.97–20.95)	16.69 (9.73–28.63)	4.85 (2.63–8.96)	34.68 (6.10–197.22)	10.45 (2.63–41.45)	0.003
**Hb (g/dl)**	10.62±2.08	10.18±1.00	10.31±1.40	9.07±1.24	10.90±2.30	9.44±1.39	10.39±1.87	0.008
**Platelet count (n. cell×10^9^/l)**	165.35±99.01	411.78±283.70	270.84±641.14	91.53±66.38	122.57±71.82	82.57±63.47	35.86±32.58	0.051
**LDH** * **(U/l)**	321 (258–399)	304 (255–362)	371.85 (284–488)	280 (227–346)	283 (243–329)	403 (156–1042)	430 (128–1441)	0.393
**NTBI (µM)**	0.01±1.15	1.59±1.96	−0.32±1.25	0.03±1.55	0.65±1.82	0.19±1.24	0.60±1.92	0.058
**s-Iron (µg/dl)**	117±53	153±69	117±55	131±64	159±67	126±55	114±43	0.441
**s-Transferrin (g/l)**	212±38	180±29	209±46	191±38	191±41	209±28	203±50	0.219
**Transferrin saturation (%)**	41.7±23.4	68.4±28.6	44.4±23.9	51.9±28.2	71.7±35.2	48.9±26.9	45.7±26.1	0.048
**GDF-15** * **(pg/ml)**	3852 (2608–5687)	4793 (2144–10714)	4630 (2408–8904)	4846 (3284–7151)	5636 (1905–16677)	3938 (2225–6972)	3971 (1161–13585)	0.976
**EPO** * **(U/l)**	37.50 (14.56–96.54)	134.24 (51.61–349.15)	82.62 (35.96–189.62)	232.87 (149.55–362.64)	187.82 (46.04–766.24)	88.46 (17.75–440.76)	83.89 (11.29–623.34)	0.011
**IPSS (%)**								
**low**	63.0	100.0	61.1	0.0	85.7	50.0	33.3	
**int-1**	33.3	0.0	38.9	34.5	14.3	33.3	33.3	
**int-2**	3.7	0.0	0.0	51.7	0.0	16.7	33.3	<0.001
**high**	0.0	0.0	0.0	13.8	0.0	0.0	0.0	
**Transfused patients (%)**	29.6	33.3	27.8	56.3	33.3	57.1	50.0	0.303

°: by Analysis of Variance (ANOVA) or by χ2-analysis, when indicated.

*: variables not normally distributed are expressed as geometric means with 95% CIs.

### Homeostatic control of hepcidin by iron

**Figure 1:**
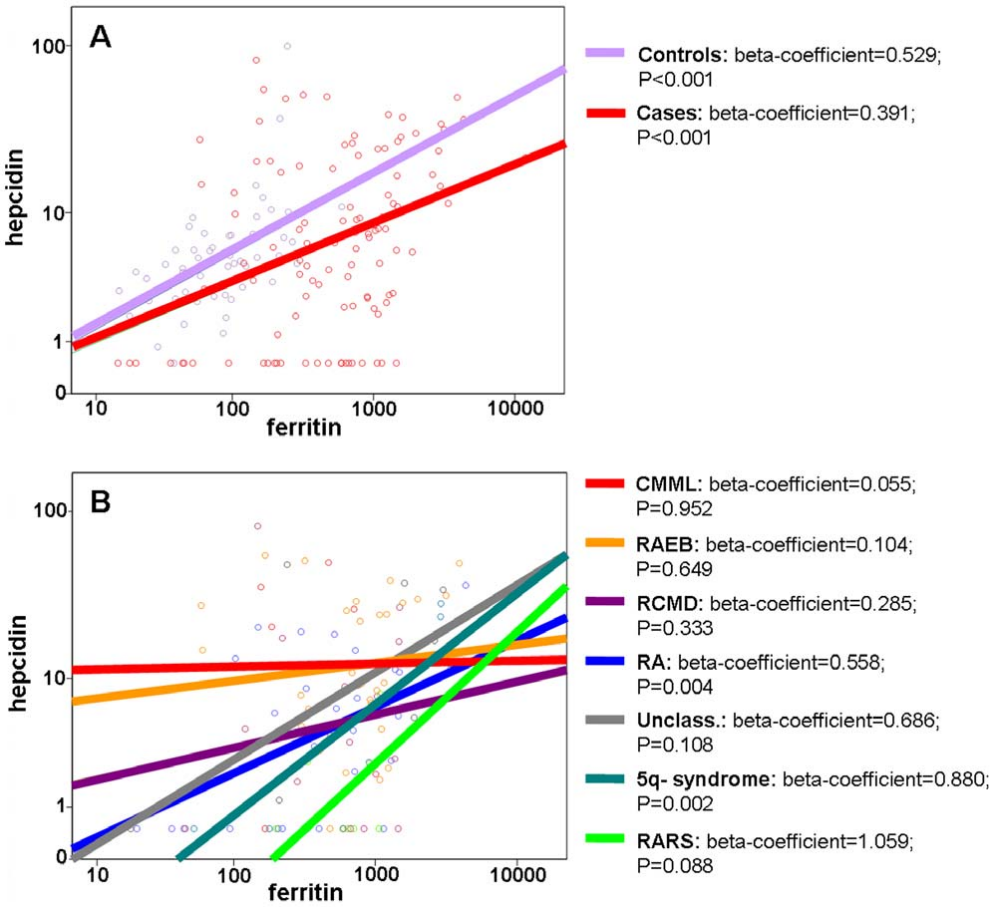
Correlation plot between hepcidin and serum ferritin levels (logarithmic scale). (A) Controls versus all MDS patients. (B) MDS patients stratified according to WHO classification.

**Figure 2:**
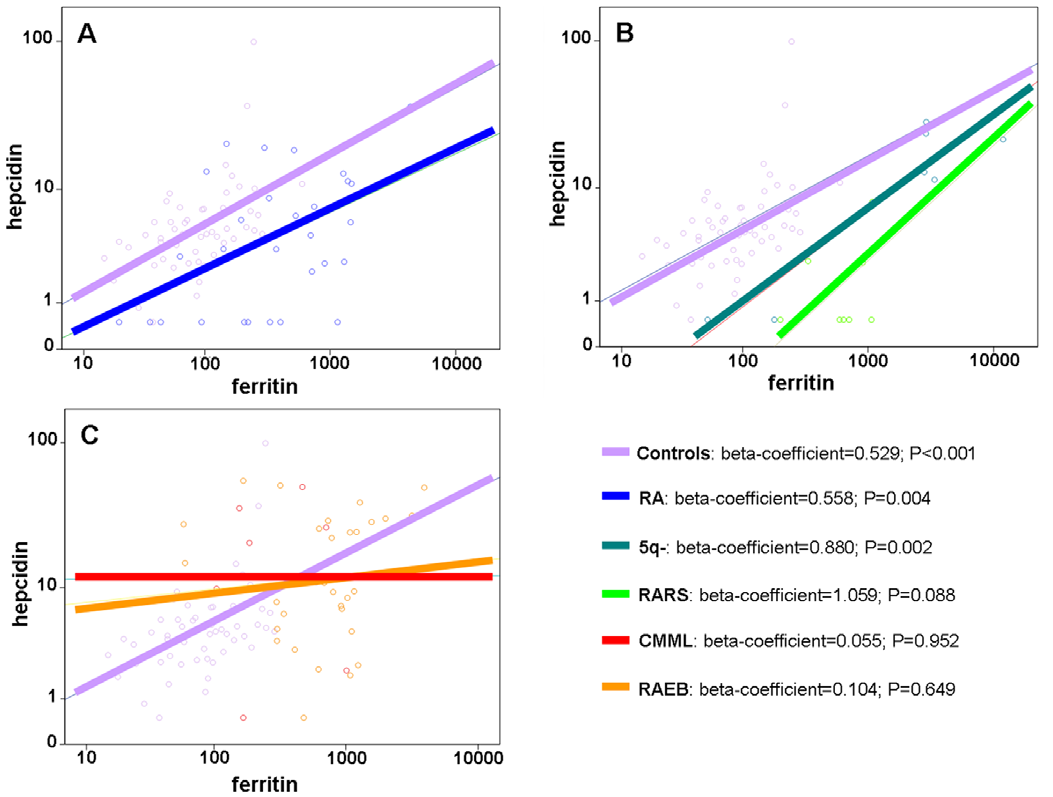
Correlation plot between serum hepcidin and serum ferritin levels in different MDS subtypes as compared to controls (logarithmic scale). (A) Controls versus RA. (B) Controls versus 5q- syndrome and RARS. (C) Controls versus CMML and RAEB.

To explore the degree of preservation of the homeostatic control of hepcidin by iron, we performed a set of general linear models. As shown in Figure 1A, when considering the MDS population as a whole, the positive correlation between hepcidin and ferritin was relatively conserved, though the hepcidin/ferritin ratio was lower than in controls suggesting a relatively blunted response. However, when MDS patients were stratified according to the different WHO subtypes a marked heterogeneity of slopes was evident (Figure 1B). This suggested the relative preservation of the homeostatic control by iron in certain MDS subtypes like RA (Figure 2A), RARS and the 5q- syndrome (Figure 2B), as well as the near complete loss of this mechanism in other MDS subtypes like RAEB and CMML (Figure 2C). Noteworthy, comparing the extremes of such models, i.e. RAEB and CMML versus RARS and 5q-, a significant difference in hepcidin/ferritin slopes was found (F = 8.684; P = 0.005).

### Hepcidin determinants in MDS patients

To evaluate the independent determinants of serum hepcidin levels in MDS patients we performed multivariate linear regression models including possible confounders like age, gender, and transfusion history (Table 3). Of note, serum ferritin levels remained as significant predictors of serum hepcidin levels, as did MDS subtypes. More precisely, when compared with RA (considered as reference subtype), RARS (with a negative β-coefficient), RAEB, and CMML (with a positive β-coefficient) were independent predictors of serum hepcidin levels (Table 3, model 1). When CRP levels were added to the model, they also were significant independent predictors of hepcidin levels along with ferritin, while the MDS subtypes with high CRP levels (RAEB and CMML) were no longer significant predictors, and the RARS subtype remained significantly associated with hepcidin (Table 3, model 2).

**Table 3:** Predictors of hepcidin levels in different linear regression models in MDS patients.

	Model 1 §		Model 2 ∧	
	β-coefficient	*P*	β-coefficient	*P*
**Female sex**	0.474	0.129	0.408	0.227
**Age (years)**	0.023	0.135	0.019	0.226
**Ferritin (µg/l)**	0.450	<0.001	0.451	0.002
**Myelodysplastic syndrome subtype** *				
**RARS**	−1.245	0.019	−1.181	0.024
**RCMD**	0.165	0.684	−0.152	0.718
**RAEB**	1.015	0.008	0.711	0.087
**5q- syndrome**	0.321	0.616	0.205	0.746
**CMML**	1.282	0.028	0.784	0.235
**Unclass.**	0.502	0.367	0.270	0.648
**Blood transfusion**	−0.181	0.538	0.066	0.830
**CRP (mg/l)**			0.210	0.020

*: considering RA as the reference group.

§ : linear regression model adjusted for sex, age, ferritin levels, MDS type and history of blood transfusion (model 1).

∧ : linear regression model adjusted for the above mentioned factors and CRP levels (model 2).

## Discussion

**Figure 3:**
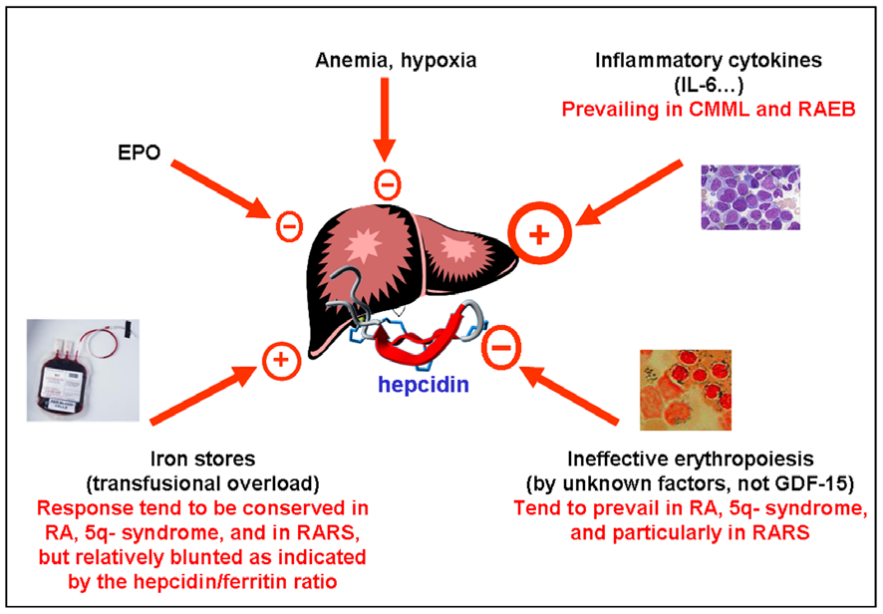
Proposed mechanisms controlling hepcidin production in different MDS subtypes.

Although percentage of marrow blasts, cytogenetic abnormalities, and cytopenias remain the prognostic cornerstones in MDS, recent data point to iron overload as an important contributing factor. Transfusion dependency have been introduced in the WHO based prognostic score [3], and serum ferritin levels have been associated to either overall or leukemia-free survival [25]. Beyond the classic detrimental effect of cardiac siderosis [26], other iron overload-related mechanisms have been proposed, including an increased risk of infections, adverse effects on hematopoietic stem cell transplantation, and a pro-oxidative state promoting genomic instability and leukemic transformation [6]. Thus, elucidating the pathophysiology of iron overload beyond the obvious role of RBC transfusions represents a relevant issue in MDS patients, with possible therapeutic implications in selecting those patients that may benefit at most from iron chelation therapy [27]–[29]. As a general rule, an important factor in determining iron toxicity is represented by the route by which the element enter the body, which in turn is unable to excrete excess iron. The parenteral route, i.e. through RBC transfusions, leads to prominent macrophage iron overload that tends to be better tolerated than the intestinal route, leading to prominent overload in periportal hepatocytes and thereafter in other parenchymal cells [30]. Given its pivotal role in orchestrating both iron absorption and recycling from macrophages, hepcidin has been an attractive candidate for studying perturbed iron homeostasis in MDS, but few and contradictory data have been available until now. Winder and colleagues [14] studied 16 MDS patients (4 RA, 3 RARS, 3 RCMD, and 6 RAEB) 13 of them chronically transfused and found undetectable or inappropriately low urinary hepcidin in most of them. These Authors suggested that hepcidin suppression through increased erythropoietic drive, and the ensuing increased iron absorption may be generalized phenomena in MDS. Murphy and colleagues [15] were unable to confirm these data in 17 low grade MDS patients (8 transfusion dependent and 7 treated with EPO), most of them showing normal, if not increased, urinary hepcidin levels. Besides the very limited patients series, both these studies suffered from methodological drawbacks, since they employed first generation semi-quantitative assays of urinary hepcidin that have been abandoned because of insufficient precision [13]. To the best of our knowledge this is the largest study on hepcidin levels in MDS conducted so far. Moreover, it takes advantage from the use of a validated quantitative MS-based assay [19], recently further improved [20]. Contrary to the prior hypothesis of a generalized hepcidin suppression, the main message from our data is that hepcidin production in MDS is consistently heterogeneous, a condition that appears to parallel the clinical and pathological heterogeneity of MDS by themselves. This is also in agreement with *in vitro* experiments showing a marked variability of sera from MDS patients in their ability to suppress hepcidin in a hepatocyte cell line [31]. The spectrum of hepcidin levels varied broadly from conditions with mean levels less than a half of those in controls, like RARS, to other ones with mean levels more than twice of controls, like RAEB and CMML (Table 2). As regards to the homeostatic control of hepcidin by iron, a similar heterogeneity was evident. Although the hepcidin/ferritin ratio showed a generalized trend toward a relatively inappropriate response, the homeostatic control by iron appeared relatively conserved in MDS subtypes generally considered at low risk (like RA, RARS and the 5q- syndrome), while it appeared almost completely lost in conditions prominent dysmyelopoiesis like RAEB and CMML. Since multivariate analyses showed that CRP was also an independent determinant of hepcidin levels in MDS along with ferritin and MDS subtypes, we could hypothesize the observed hepcidin heterogeneity as the result of the relative strength of opposing stimuli in different clinical and pathological conditions (Figure 3). The main actors in this sense may be represented by the suppressing effect from ineffective erythropoiesis, variably counterbalanced by the stimulating effects from either increased iron stores or cytokines, of whom CRP is a surrogate measure. RARS may represent the prototype of MDS where the inhibition from the erythropoietic drive tends to prevail, only partially balanced by the RBC transfusions, either directly or indirectly through increased iron stores. This condition, characterized by the lowest hepcidin/ferritin ratio, indeed showed also the highest values of biochemical iron parameters indicating both an expansion of the plasma iron pool through increased absorption/recycling and parenchymal iron toxicity, like transferrin saturation [32], [33] and NTBI. Of note, studies from Mariani and colleagues on hepatic hepcidin mRNA in two RARS patients showed low levels consistent with this view [34]. At the other end of the spectrum lies RAEB and CMML, where the highest levels of both hepcidin/ferritin ratio and CRP may mirror hepcidin stimulation through blast-derived cytokines that overcomes controls by iron. Consistently with this hypothesis, both RAEB and CMML lost their significant predictivity on hepcidin level in a multivariate model adjusted with CRP levels. In this condition, the relative excess of hepcidin could favour iron entrapment within macrophages, limiting toxicity due to uncontrolled release of the element into the plasma and redirection to parenchymal cells.

As regards to the hepcidin suppression from the erythropietic drive, our results argue against a role of GDF-15 as the putative mediator of this biological effect in MDS, at variance with what observed in thalassemic syndromes [10]. GDF-15, also known as bone morphogenetic protein (BMP) 14, is a secreted morphogen of the transforming growth factor-beta super-family, conferring signaling by activation of Smad 1/5/8 or mitogen-activated protein kinase (p38-MAPK) [35]. It is highly expressed by erythroid precursors in conditions of ineffective erythropoiesis, but barely detectable in normal bone marrow [11]. In our series, which again is the largest so far evaluating GDF-15 in MDS, mean serum levels of this protein were near six to ten-fold higher than reference values, with a relative homogeneity across different MDS subtypes. Ramirez et al. [36] measured serum GDF-15 in a specific study limited to twenty RARS patients, finding similar levels (3254±1400 ng/ml) to our RARS series. Of note, this fascinating and pleiotropic biomarker has been consistently associated also to cardiovascular diseases in recent studies [37], [38], an issue that might merit further consideration in the future within the specific context of MDS. Nevertheless, GDF-15 was not correlated at all with hepcidin levels in our series. The apparent discrepancy of our results with those of Tanno and co-workers in thalassemia [10] may be explained in terms of absolute levels. Indeed, the GDF-15 levels reported in thalassemic patients are consistently higher (up to more than 100,000 pg/ml) than those found in our MDS series (mean levels near 4,500 pg/ml), and *in vitro* studies have shown that significant hepcidin suppression requires very high levels, i.e. no less than 5,000 pg/ml, being still incomplete at the highest dose of 100,000 pg/ml [10]. Recent expression studies in erythroblasts [39] have shown that erythroid regulation of hepcidin may be an heterogeneous phenomenon mediated by other molecules, i.e. TWSG1 (Twisted Gastrulation) for which serum assay is not yet available. Further studies are needed to clarify which mediators may play a role in hepcidin suppression at least in certain MDS subtypes, particularly in RARS [34]. The observation that iron biochemical parameters are significantly higher than in controls also in our subset of non transfused patients (Table S1), also reported by others [27] is a further argument in favour of a certain degree of iron hyperabsorption in MDS.

Our study suffers of several limitations that need to be acknowledged. First, our considerations on hepcidin regulation by iron rely on ferritin levels, which are known to be an imperfect marker of iron stores [40]. Other measures of body iron stores such as liver iron content (LIC) through Magnetic Resonance (MR) [41], [42] may be more accurate, considering that the “gold standard” represented by liver biopsy is clearly unfeasible in thrombocytopenic and generally elderly patients with several comorbidities like those with MDS. Nevertheless, recent data by Armand and colleagues [43] indicate that serum ferritin is still an acceptable marker of iron stores in MDS, since it showed a strong and significant correlation (r = 0.75, P<0.001) with estimated LIC by MR. Similarly, although our hepcidin assay is specific for the 25-mer bioactive isoform and has been clinically validated in other settings [19], [20], [44], [45], we have to recognize that we still lack a gold standard for measuring this hormone in biological fluids [12]. Finally, the effect of inflammatory cytokines [46], [47], which may play a prominent role in certain MDS subtypes with excess myeloblast activation, could be studied only indirectly, through a surrogate like CRP.

Notwithstanding these limitations our results, if confirmed, may be relevant for a better understanding of iron pathophysiology in MDS. They point toward an heterogeneity that, like in any one physiological situation, is determined by the relative strengths of competing stimuli in different MDS subtypes (Figure 3), with possible implications also at the individual level. This may help to calibrate possible future therapeutic approaches in MDS patients with either iron chelators [6], [29] or hepcidin modulators [48], [49].

## Supporting Information

Figure S1 **Mean levels of serum hepcidin, serum ferritin, and hepcidin/ferritin ratio across different MDS subtypes.** * : for hepcidin/ferritin ratio, P<0.001 by ANOVA with polynomial contrasts for linear trend.
(TIF)

Table S1 **Biochemical parameters of the MDS patients (either as whole population or stratified into transfused or non-transfused groups) as compared to sex-matched healthy controls.**
(DOC)

Table S2 **Clinical and biochemical characteristics of MDS patients stratified according to the IPSS.**
(DOC)
