# Supplementary figures and images for: Hepcidin Levels and Their Determinants in Different Types of Myelodysplastic Syndromes
Source: PLoS One. 2011 Aug 19;6(8):e23109. doi: 10.1371/journal.pone.0023109 (PMC3158762; doi:10.1371/journal.pone.0023109)

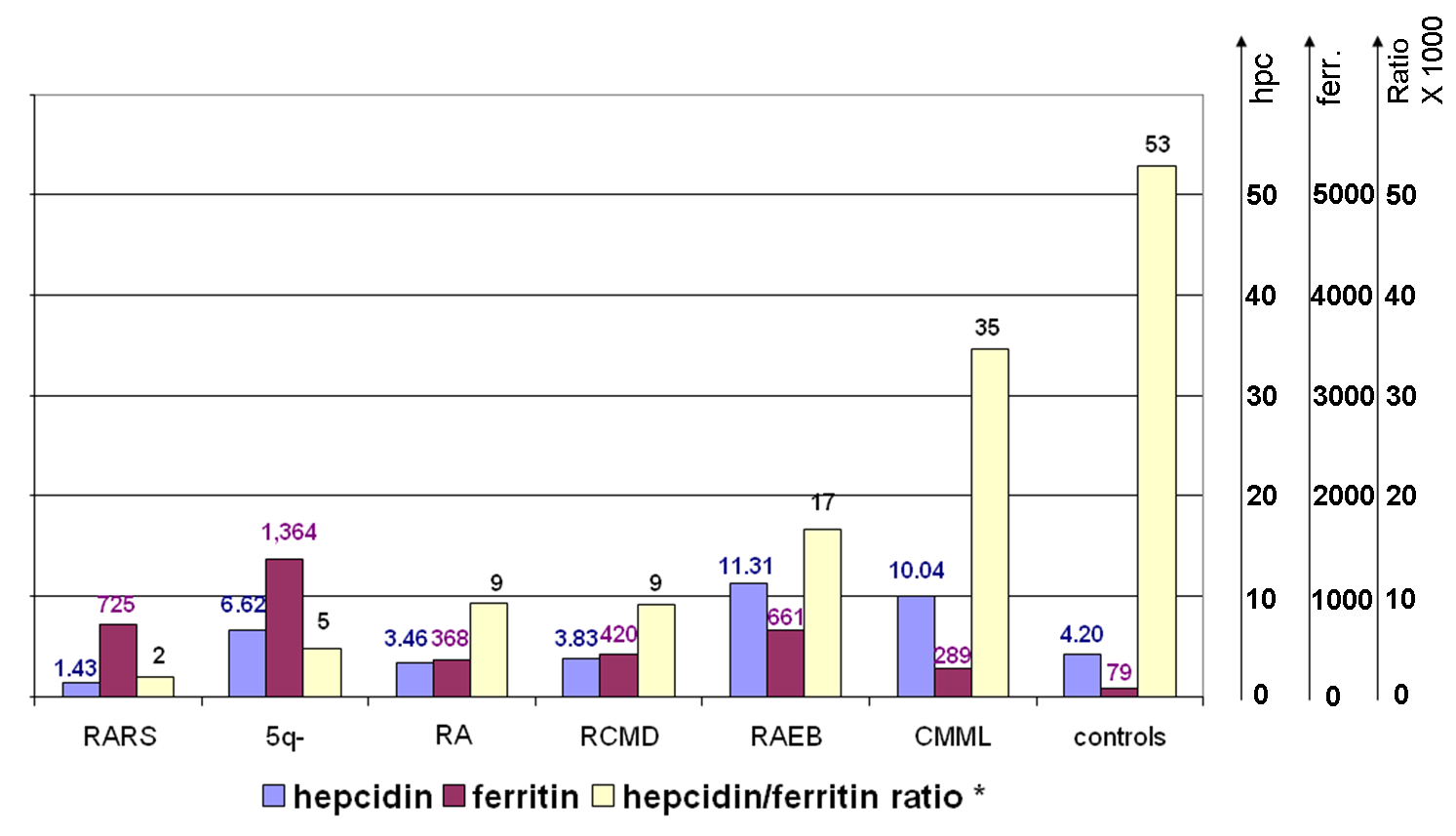

Supplement: Figure S1 — Mean levels of serum hepcidin, serum ferritin, and hepcidin/ferritin ratio across different MDS subtypes. * : for hepcidin/ferritin ratio, P<0.001 by ANOVA with polynomial contrasts for linear trend. (TIF) [file pone.0023109.s001.tif]
