# Supplementary material for: Hepcidin Levels and Their Determinants in Different Types of Myelodysplastic Syndromes
Source: PLoS One. 2011 Aug 19;6(8):e23109. doi: 10.1371/journal.pone.0023109 (PMC3158762; doi:10.1371/journal.pone.0023109)
Supplement: Table S1 — Biochemical parameters of the MDS patients (either as whole population or stratified into transfused or non-transfused groups) as compared to sex-matched healthy controls. (DOC) [file pone.0023109.s002.doc]

**Table S1.** Biochemical parameters of the MDS patients (either as whole population or stratified into transfused or non-transfused groups) as compared to sex-matched healthy controls.

|  | **Controls (n=54)** | **Whole MDS population (n=113)** | **MDS non-transfused (n=63)** | **MDS transfused (n=44)** | **P^1^** | **P^2^** | **P^3^** | **P^4^** |
| --- | --- | --- | --- | --- | --- | --- | --- | --- |
| **Age (years)** | 34.8 ± 15.8 | 72.8 ± 9.2 | 72.7 ± 8.4 | 72.7 ± 10.2 | <0.001 | <0.001 | <0.001 | 0.980 |
| **Male Sex (%)** | 61.1 | 68.1 | 60.3 | 81.8 | 0.370 | 0.541 | 0.021 | 0.014 |
| **Hemoglobin (g/dl)** | n.a. | 10.01 ± 1.73 | 10.88 ± 1.50 | 8.71 ± 1.25 | n.e. | n.e. | n.e. | <0.001 |
| **s-iron ( µg/dl)** | 100 ± 28 | 127 ± 59 | 106 ± 49 | 161 ± 58 | <0.001 | 0.375 | <0.001 | <0.001 |
| **Transferrin ( g/l)** | 251± 37 | 201± 40 | 208 ± 38 | 185 ± 35 | <0.001 | <0.001 | <0.001 | 0.002 |
| **Transferrin saturation (%)** | 28.9 ± 9.1 | 49.8 ± 27.4 | 40.8 ± 23.9 | 66.0 ± 26.1 | <0.001 | <0.001 | <0.001 | <0.001 |
| **Ferritin ( µg/l)** | 79 (64-97) | 515 (407-652) | 392 (285-538) | 925 (694-1232) | <0.001 | <0.001 | <0.001 | <0.001 |
| **Hepcidin (nmol/l)** | 4.20 (3.53-5.00) | 5.31 (3.98-7.08) | 4.13 (2.81-6.06) | 9.08 (5.95-13.84) | 0.288 | 0.938 | <0.001 | 0.007 |
| **Hepcidin/ferritin ratio (nmol/µg x 1000** | 52.94 (43.57-64.33) | 10.10 (7.53-13.53) | 10.54 (7.12-15.62) | 9.37 (5.67-15.50) | <0.001 | <0.001 | <0.001 | 0.710 |
| **CRP (mg/l)** | 1.07 (0.93-1.23) | 3.81(2.71-5.36) | 2.89 (1.82-4.57) | 5.62 (3.30-9.54) | <0.001 | <0.001 | <0.001 | 0.062 |
| **NTBI(µM)** | n.a. ()* | 0.18 ± 1.51 | -0.21 ± 1.42 | 0.83 ± 1.51 | n.e. | n.e. | n.e. | <0.001 |
| **GDF-15 (pg/ml)** | n.a. ()* | 4422 (3591-5445) | 3330 (2700-4106) | 6116 (4175-8961) | n.e. | n.e. | n.e. | 0.003 |
| **EPO (U/l)** | n.a. ()* | 102.49 (72.15-145.61) | 60.43 (36.02-101.37) | 240.88 (171.30-338.73) | n.e. | n.e. | n.e. | <0.001 |

n.a.: not assessed in this control population.

n.e.: not evaluable

* reference value in healthy individuals according to the manufacturer’s protocol

NTBI (µM): -0.72 ± 0.39

GDF-15 (pg/ml): 641 (401-881)

EPO (U/l): 3.7-37.5

P^1^ = whole MDS population versus controls

P^2^ = non-transfused MDS patients versus controls

P^3^ = transfused MDS patients versus controls

P^4^ = non-transfused MDS patients versus transfused MDS patients
