# Supplementary material for: Hepcidin Levels and Their Determinants in Different Types of Myelodysplastic Syndromes
Source: PLoS One. 2011 Aug 19;6(8):e23109. doi: 10.1371/journal.pone.0023109 (PMC3158762; doi:10.1371/journal.pone.0023109)
Supplement: Table S2 — Clinical and biochemical characteristics of MDS patients stratified according to the IPSS. (DOC) [file pone.0023109.s003.doc]

**Table S2.** Clinical and biochemical characteristics of MDS patients stratified according to the IPSS.

|  | **Low (n=48)** | **Int-1 (n=31)** | **Int-2 (n=19)** | **High (n=4)** | **P^1^** | **P^2^** |
| --- | --- | --- | --- | --- | --- | --- |
| **Age (years)** | 73.71 ± 9.00 | 74.00 ± 11.03 | 69.32 ± 5.95 | 66.50 ± 8.43 | 0.142 | 0.050 |
| **Male sex (%)** | 58.3 | 74.2 | 94.7 | 50.0 | 0.022 | 0.036 |
| **CRP* (mg/l)** | 1.87 (1.04-3.39) | 4.92 (2.88-8.40) | 9.02 (4.66-17.46) | 5.08 (0.07-385.64) | 0.006 | 0.001 |
| **Ferritin* (µg/l)** | 424 (279 -645) | 570 (370-878) | 910 (680-1217) | 144 (19-1097) | 0.044 | 0.256 |
| **Hepcidin* (nmol/l)** | 3.47 (2.24-5.38) | 4.17 (2.33-7.49) | 10.67 (6.70-16.99) | 21.51 (2.63-175.97) | 0.007 | 0.001 |
| **Hepcidin/Ferritin* Ratio**  **(nmol/µg x 1000)** | 8.18 (5.32-12.59) | 7.32 (4.18-12.82) | 12.37 (7.89-19.39) | 102.19 (1.17-8953.50) | 0.018 | 0.038 |
| **Hb (g/dl)** | 10.74 ± 1.41 | 9.76 ± 2.01 | 9.05 ± 1.15 | 8.95 ± 2.00 | 0.001 | < 0.001 |
| **Platelet count (n. cell x 10^9^/l)** | 271.43 ± 428.46 | 85.43 ± 48.90 | 102.11 ± 74.46 | 40.00 ± 26.27 | 0.028 | 0.009 |
| **LDH* (U/l)** | 339 (292-394) | 316 (261-381) | 294 (215-402) | 224 (126-397) | 0.337 | 0.090 |
| **NTBI (µM)** | 0.09 ± 1.50 | 0.44 ± 1.41 | -0.04 ± 1.59 | -0.35 ± 1.93 | 0.593 | 0.732 |
| **s-Iron ( µg/dl)** | 124 ± 59 | 137 ± 60 | 130 ± 59 | 57 ± 33 | 0.158 | 0.688 |
| **s-Transferrin (g/l)** | 205 ± 42 | 202 ± 40 | 193 ± 37 | 176 ± 63 | 0.508 | 0.166 |
| **Transferrin saturation (%)** | 49.3 ± 28.6 | 53.2 ± 25.2 | 52.7 ± 29.7 | 20.7 ± 6.5 | 0.265 | 0.709 |
| **GDF-15* (pg/ml)** | 4017 (2869-5625) | 4236 (3015-5953) | 5949 (3174-11152) | 3104 (403-23880) | 0.575 | 0.430 |
| **EPO* (U/l)** | 54.09 (28.17-103.86) | 124.17 (72.60-212.36) | 186.68 (90.33-385.79) | 333.89 (121.21-919.75) | 0.018 | 0.002 |
| **DIAGNOSIS(%)** |  |  |  |  |  |  |
| **RA** | 35.4 | 29.0 | 5.3 | 0.0 |  |  |
| **RARS** | 18.8 | 0.0 | 0.0 | 0.0 |  |  |
| **RCMD** | 22.9 | 22.6 | 0.0 | 0.0 |  |  |
| **RAEB** | 0.0 | 32.3 | 78.9 | 100.0 |  |  |
| **5q- syndrome** | 12.5 | 3.2 | 0.0 | 0.0 |  |  |
| **CMML** | 6.3 | 6.5 | 5.3 | 0.0 |  |  |
| **Unclass** | 4.2 | 6.5 | 10.5 | 0.0 |  |  |
| **Transfused patients (%)** | 20.9 | 56.7 | 52.6 | 50.0 | 0.010 | 0.008 |

P^1^ = by ANOVA or by χ2-analysis, when indicated

P^2^ = by ANOVA with polynomial contrasts for linear trend or by χ2-for linear trend, when indicated
